# Atrioesophageal fistula after atrial fibrillation catheter ablation: A case report

**DOI:** 10.1097/MD.0000000000024226

**Published:** 2021-01-15

**Authors:** Fan He, Wei-Min Zhang, Bi-Jun Xu, Gang-Ping Huang, Huai-Dong Chen

**Affiliations:** Department of Cardiac Surgery, Sir Run Run Shaw Hospital, School of Medicine, Zhejiang University, Hangzhou, China.

**Keywords:** atrial fibrillation, atrioesophageal fistula, catheter ablation, surgical treatment

## Abstract

**Rationale:**

Atrioesophageal fistula (AEF) is a rare but serious complication of atrial fibrillation (AF) catheter ablation with associated high mortality rates.

**Patient concerns:**

A 42-year-old male patient who underwent catheter ablation in local hospital 20 days ago because of persistent AF was admitted to our Emergency Room with unconsciousness and high axillary temperature and white blood cell count. Craniocerebral CT scan found multiple infarct lesions in both frontal and occipital lobes. Pneumatosis between the left atrium and the esophagus was observed in the chest CT.

**Diagnoses:**

AEF.

**Interventions:**

We performed a salvage operation of the left atrium debridement, and left atrium patch repairing under extracorporeal circulation. We opened the mediastinum, and dissected the esophageal perforation. A special irrigating catheter with multiple side ports on the tip was placed from the esophagus to the posterior mediastinum through the esophageal orificium fistulae. We also inserted a gastrointestinal tube to the jejunum under gastroscopy. Three additional drainage tubes were inserted into the esophageal bed and the right thoracic cavity.

**Outcomes:**

The procedure was successful. But 7 days later, the patient's family chose to forgo treatment due to multiple cerebral infarcts, respiratory and blood system infection, liver failure, and other complications.

**Lessons:**

AEF is a rare but fatal complication after catheter ablation. Heightened vigilance is required for early recognition of the AEF. Surgical treatment should be performed as early as possible, especially before the neurological complications occur.

## 1 Introduction

Atrioesophageal fistula (AEF) is a rare but fatal complication in catheter ablation for atrial fibrillation (AF). The incidence of AEF after catheter ablation is less than 1%, but the mortality is nearly 80%.^[1,2]^ Its early symptoms mainly include fever, drowsiness, weakness, chest pain, dysphagia, nervous system symptoms, hematemesis, and melena. Furthermore, it usually occurs 1 to 6 weeks after ablation, and the average time is about 20 days after surgery.^[2,3]^ The diagnosis and early treatment of AEF is often delayed due to extensive symptoms, delayed onset, and lack of clinical awareness. The outcome after surgery is usually poor for serious neurological complications and untreatable mediastinal infection. These may explain the high mortality of this disease. Here, we report a case of AEF after catheter ablation for AF. Although we successfully performed cardiac repair and efficient esophageal drainage on the patient, unfortunately, the patient had multiple cerebral infarctions before the surgery and abandoned further therapy for multiple organ dysfunction syndrome on the 7th day after the surgery.

## 2 Case report

A 42-year-old male patient was admitted to our emergency department because of “upper abdominal pain for 4 days, cough with fever for 2 days and loss of consciousness for 9 hours”. The patient had a long history of AF. Twenty days ago, he underwent catheter ablation in the department of cardiology of local hospital. He was discharged successfully without any incident. Four days ago, the patient complained upper abdominal pain after meal that relieved on the next day. This was followed by cough and fever 2 days ago. As a consequence of not visiting the doctor in time, he suddenly lost consciousness accompanied by no response to calling, no urinary incontinence and no physical convulsion symptom. Thus he was transferred to our hospital soon after the attack.

**Figure 1:**
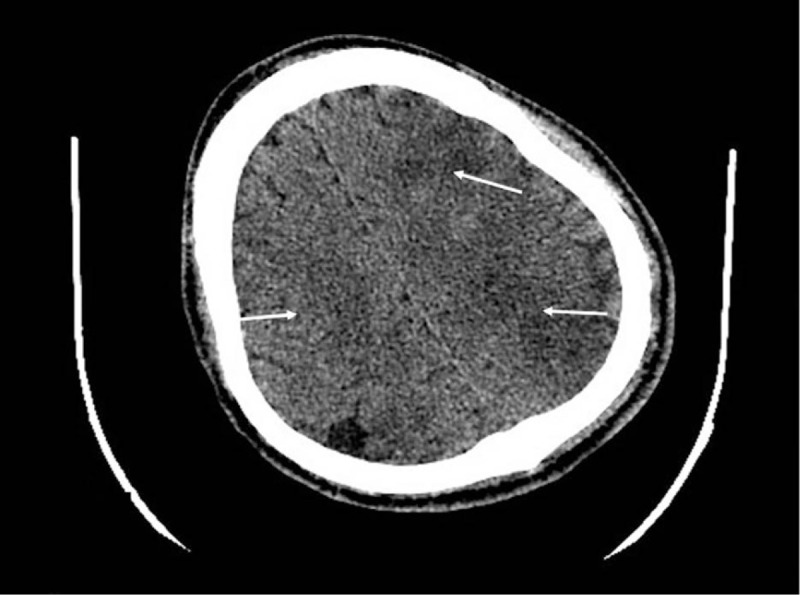
Craniocerebral CT showed multiple infarcts in both frontal and occipital lobes (as shown by the arrow).

**Figure 2:**
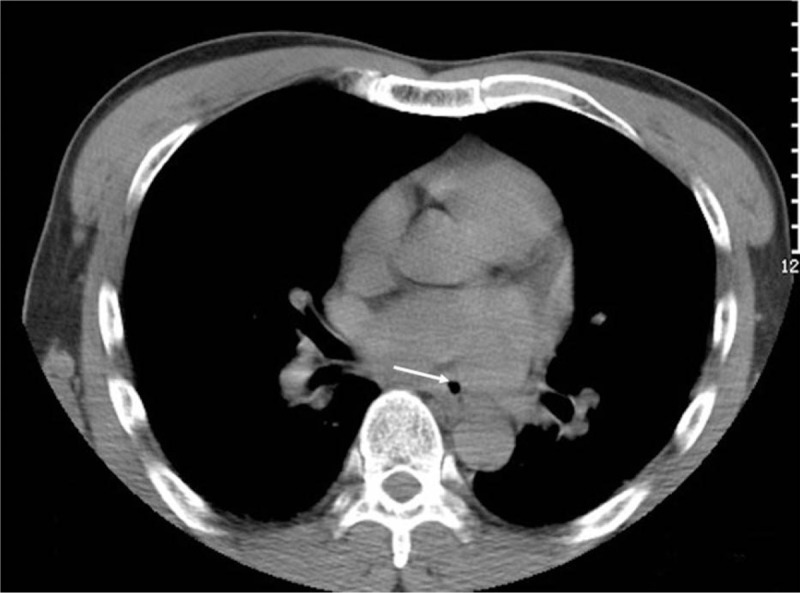
Chest CT suggested gas shadow in the middle segment of the esophagus near the left atrium, suggesting atrial esophageal fistula (as shown by the arrow).

Physical examination on admission showed that the pupils were equal, with a diameter of 3 mm, but reflected to light slowly. The body temperature was 39.8°C, heart rate 133 bpm, the blood pressure 147/79 mm Hg, and the breathing rate 30 times/min. Laboratory examinations showed that white blood cell count was 22 × 10^9^/L, procalcitonin was 35.33ng/mL, high sensitivity C reactive protein was 180 mg/L, and the myocardial enzyme profile increased significantly in the meantime. The electrocardiograph showed extensive ST segment elevation. The craniocerebral CT scan found multiple infarct lesions in both frontal and occipital lobes (Fig. 1). Meanwhile, the chest CT scan found a small amount of pneumatosis in posterior mediastinum located at the middle segment of the esophagus, close to the left atrium. Multiple inflammatory lesions in both lungs could also be seen (Fig. 2). The transthoracic echocardiography (TTE) showed no signs of infective endocarditis. The patient was diagnosed as AEF with acute myocardial infarction and acute cerebral infarction. Sepsis was highly suspected.

As the patient had fever with a significantly increased inflammatory index, we highly suspected that the blood infection might due to the atrial esophageal fistula. Since there was no blood culture result at that time, we administered empirical anti-infection treatment with meropenem. Multi-disciplinary team discussion was conducted immediately after admission, including cardiology, cardiac surgery, infection department, neurology, the department of Critical Care Medicine, and other related departments. In consideration of the patient's young age, strong willingness for treatment from his family, and the consultation opinions of relevant specialties, we finally decided to perform the salvage surgery for the patient.

**Figure 3:**
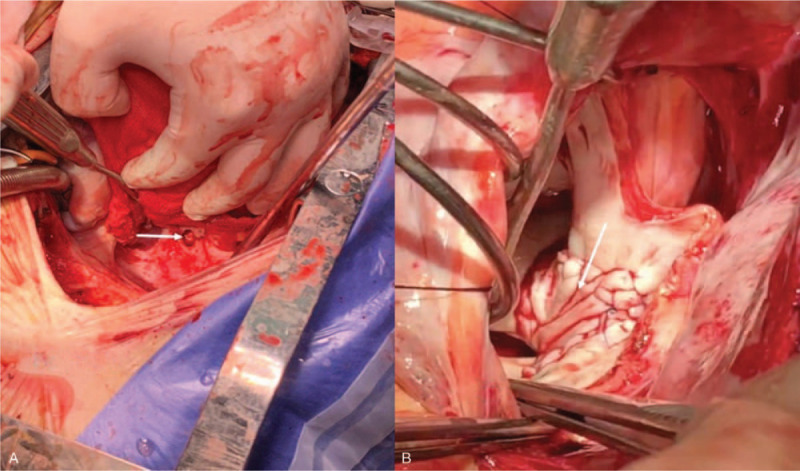
A: A crevasse of about 2∗2 cm can be seen in the posterior wall of the left atrium, a vegetation about 5∗0.5 cm in size and some thrombus adherent to the posterior wall of the left atrium were also found.; B: Bovine pericardial patch was used to repair the perforated posterior wall of the left atrium during the operation (as shown by the arrow).

**Figure 4:**
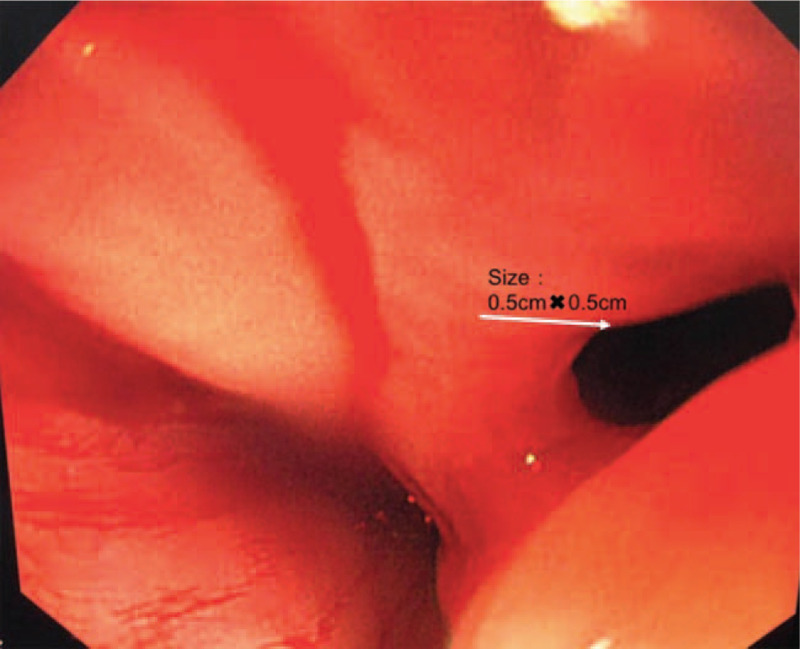
Intraoperative gastroscopy showed a 0.5 × 0.5 cm crevasse in the middle segment of the esophagus (as shown by the arrow).

The sternal midline incision was selected. After heparinization and establishing extracorporeal circulation, we clamped the ascending aorta and perfused the cardioplegia. The left atrium was opened through the interatrial groove incision. We found a hole, 2 cm in diameter on the posterior wall of the left atrium. The hole was connected with the esophagus, and some bubbles were coming outside from time to time. A vegetation about 5 × 0.5 cm in size and some thrombosis adherent to the posterior wall of the left atrium were also found (Fig. 3A). The operation was carried out in 3 steps. First, the vegetation and thrombosis in the left atrium were removed. The puncture of the posterior wall of the left atrium was repaired with a bovine pericardial patch, and the left atrial incision was sutured (Fig. 3B). Secondly, the heart was suspended with a gauze net. The unhealthy pericardium tissue closing to the fistula was removed, and the pericardium hole was sutured directly. Finally, the right lung tissue was retracted forward and upward, and the posterior mediastinal pleura was opened to dissect the perforated esophagus. A perforated irrigation catheter from the nasopharyngeal approach was placed into the esophageal bed through the esophageal fistula under the guidance of the visual gastroscopy. The size of esophageal fistula under gastroscopy was about 0.5 × 0.5 cm (Fig. 4). A gastrointestinal tube was also placed to the jejunum under the gastroscopy. A mediastinal drainage tube was indwelling in the esophageal bed beside the esophageal fistula, and 2 right thoracic drainage tubes were placed into the right thoracic cavity. The operation was successful and the patient was admitted to ICU for intensive care.

After the operation, vancomycin combined with meropenem for anti-infection therapy and idaravone for nerve nutritional therapy were given. The patient's vital signs were stable and the inflammatory indicators were significantly decreased. Unfortunately, the coagulation function and liver function were deteriorating. On the third day after surgery, the inflammatory indicators increased again. white blood cell count was 28.9∗10^9^/L and procalcitonin was 14.97ng/mL. Postoperative blood culture results revealed streptococcus/oral streptococcus. Thus the antibiotics were changed to meropenem combined with cefuroxime. The electrocardiogram indicated that ST-segment elevation of multiple leads, and Troponin I was more than 30ng/mL. TTE indicated moderate mitral valve insufficiency accompanied by severe tricuspid valve insufficiency, and the left ventricular ejection fraction was 30%, which was considered to be myocardial ischemia and papillary muscle dysfunction. In the followed days, the patient's condition continued to deteriorate, including heart failure, enlarged cerebral infarction area, aggravated infection in lungs and blood, decreased blood pressure, abnormal coagulation function, thrombocytopenia, and abnormal liver and kidney functions. Despite aggressive treatment, postoperative infection could not be controlled effectively. The patient's family finally gave up treatment due to multiple organ dysfunction disorder on day 7 after surgery.

## 3 Discussion

Catheter ablation is an effective method to treat AF. Though the incidence of AEF post the procedure of catheter ablation is less than 1%, the mortality is nearly 80%.^[1,2]^ A survey involving 190,000 patients with catheter ablation for AF showed that 79% of patients died from AEF.^[4]^ Due to the inconspicuous early symptoms and the diversity of late symptoms, it is most likely misdiagnosed or the diagnosis is missed. This may be the cause for high mortality. For most patients, the early symptoms of AEF are not obvious. It usually includes neurological symptoms such as changes in mental status, epilepsy, hemiplegia, and stroke. Other symptoms include fever, lethargy, weakness, chest pain, difficulty swallowing, vomiting blood, or melena.^[2]^ These symptoms become apparent 1 to 6 weeks after procedure. Unfortunately, AEF is often misdiagnosed as “cold”, gastritis, cerebral infarction, and other diseases, because patients usually lack medical vigilance and cardiologists have limited experience with this disease. The patient did not realize the fatal danger even when he had abdominal pain, fever and cough which appeared 2^+^ weeks later after ablation. He had considered it was just a “cold” until it was too late and he lost consciousness. Therefore, heightening vigilance is probably the most important thing in early recognition of AEF.

The exact mechanism of AEF post procedure of catheter ablation is not very clear. An esophageal injury (a coagulative necrosis) during catheter ablation is the trigger event, followed by further mechanical damage by big rough block food or chemical inflammation by gastric reflux, which could result in a continuous esophageal injury, perforation, and fistula formation. Obvious symptoms appear around 20 days after the catheter ablation, while some patients may have this complication as early as 6 days after ablation and some as late as 59 days.^[5]^ The delayed onset of AEF after catheter ablation suggests that mechanical perforation of atrial wall during ablation is unlikely to be responsible. Most experts believe that AEF is related to the close anatomic distance between the left atrial posterior wall and the middle segment of the esophagus. The radiofrequency energy might cause damage to the esophageal mucosa during the procedure in all probability. Especially in patients with large left atrium, a thin posterior wall, and an enlarged contact area between esophagus and the left atrium, it is more likely to arise as a risk of esophageal injury.^[6]^ Once the AEF occurs, large amount of blood passes through the fistula from atrium to esophagus. The gastrointestinal bleeding, melena, and other symptoms consequently appear. On the other hand, esophageal peristalsis can increase the esophageal pressure to more than 10 times over the atrial pressure. This facilitates the air bubbles and esophageal contents to enter the left cardiac system, and leads to sepsis and multiple organ infarctions (such as myocardial infarction, cerebral infarction, etc.^[7,8]^

It is difficult to diagnose AEF timely and effectively especially when transesophageal echocardiography and esophagogastroduodenoscopy (EGD) are relative contraindicated. On 1 hand, air may be carried into the brain, the coronary artery system and other organs during the examination, and may cause embolism; On the other hand, the placement of EGD can promote esophageal peristalsis, which might expand the esophageal fistula.^[6,9]^ The carbon dioxide can be used for avoiding the risk of air embolism, if using EGD to diagnose and estimate the size of damaged esophageal wall and the presence of bleeding ulcer.^[10]^ For AEF patients, chest CT scan can safely provide the necessary anatomical information.^[6]^ However, some doctors believe that conventional chest CT may not provide accurate diagnosis due to slight abnormalities of left atrium or the whole heart, but enhanced CT is more meaningful for the diagnosis of AEF.^[9]^ For our case, due to the late onset, the symptoms and signs were obvious. The plain chest CT scan in our hospital showed low-density gas shadow in the posterior wall of the left atrium. Therefore, we believe that plain chest CT scan can be a basic examination for patients with highly suspected AEF. For AEF that cannot be identified by conventional chest CT, chest or cardiac enhanced CT is recommended. Gastroenteroscopy can diagnose the disease definitely, but considering the risk of air embolism, we do not recommend it as a routine diagnostic method. However, in the process of esophageal surgery after cardiac repair, it can assist in locating the esophageal perforation, as well as helping to place the esophago-mediastinal irrigation tube and the gastroduodenal nutrition tube. Considering the pathophysiological changes of AEF, TTE is not specific for its diagnosis. But it should still be used as an auxiliary examination. Since there is no specific early diagnostic examination for AEF, we suggest C reactive protein should be monitored every week after interventional catheter ablation. Patients should be followed up closely in the first 4 weeks after the surgery.

At present, there are 2 treatment options for AEF patients: the conservative treatment and the surgical treatment. Conservative treatment includes using broad-spectrum antimicrobial drugs and respiratory support by mechanical ventilation. Antibacterial drugs usually include vancomycin, piperacilin-tazobactam, meropenem, and fluconazole.^[11]^ There have been a few successful reports of non-surgical treatments such as esophageal stent implantation and pericardiocentesis. These are usually the cases where the esophagus suffers little thermal damage and scars have formed.^[12]^ However, stenting increases the risk of air embolism.^[13]^ Regardless of surgical intervention, active prophylactic therapy with proton pump inhibitors can theoretically reduce the risk of AEF formation, but evidence is lacking. For neurological sequelae, early use of high pressure oxygen has been described, but no specific data has confirmed its effectiveness.^[14]^ Surgical treatment can improve the AEF patients’ survival rate. The importance of primary esophageal repair and left atrial repair to reduce the postoperative complications has been previously demonstrated.^[15]^ Houman Khakpour et al reported their method where they used a patch to repair the posterior wall of the left atrium, separated the distal esophagus from the posterior wall of the left atrium, covered the esophageal fistula with the right intercostal muscle, and sutured the right lats muscle to the repair site of the left atrium. These procedures could completely separate the left atrium from the esophagus, and the pedicled muscle flap could also provide blood supply to tissue in this area.^[11]^

In our case, we reconstructed the posterior wall of the left atrium with a bovine pericardium patch and closed the perforated pericardium directly. To treat the perforated esophagus, we adopted the same method of treatment of anastomotic fistula after general thoracic esophageal surgery where the perforated irrigation tube is interposed across the fistula for washing, and drainage tubes are interposed beside the esophagus and in the right chest. This method has achieved good results in the cases of esophageal fistula.^[16]^ As long as the irrigation and drainage are unobstructed, esophageal fistulae can self-heal. The patient in this report expired due to postoperative hemodynamic instability caused by myocardial infarction, progressive deterioration of cerebral infarction lesions, and recurrent bloodstream infection. For patients with such hemodynamic instability, extracorporeal membrane oxygenation (ECMO) support after surgery may provide a time window for cardiopulmonary function recovery, but bloodstream infection is a relative contraindication to ECMO.

In conclusion, AEF is a rare but fatal complication after catheter ablation. Heightened vigilance is probably the most important thing in early recognition of the AEF. Surgical repair should be performed as early as possible, especially before the neurological complications occur.

For patients with postoperative hemodynamic instability and cardiopulmonary insufficiency, ECMO could be an important transitional measure to prevent further deterioration of the patient's condition.

## Author contributions

**Conceptualization:** Huai-Dong Chen.

**Methodology:** Gang-Ping Huang.

**Supervision:** Bi-Jun Xu.

**Visualization:** Wei-Min Zhang.

**Writing – original draft:** Fan He.

**Writing – review & editing:** Wei-Min Zhang.

## Abbreviations

Abbreviations: AEF = atrioesophageal fistula, AF = atrial fibrillation, ECMO = extracorporeal membrane oxygenation, EGD = esophagogastroduodenoscopy, TTE = transthoracic echocardiography.
